# A Robust Approach to Identifying Tissue-Specific Gene Expression Regulatory Variants Using Personalized Human Induced Pluripotent Stem Cells

**DOI:** 10.1371/journal.pgen.1000718

**Published:** 2009-11-13

**Authors:** Je-Hyuk Lee, In-Hyun Park, Yuan Gao, Jin Billy Li, Zhe Li, George Q. Daley, Kun Zhang, George M. Church

**Affiliations:** 1 Department of Genetics, Harvard Medical School, Boston, Massachusetts, United States of America; 2 Division of Pediatric Hematology Oncology, Children's Hospital Boston, Boston, Massachusetts, United States of America; 3 Center for the Study of Biological Complexity, Virginia Commonwealth University, Richmond, Virginia, United States of America; 4 Department of Bioengineering, University of California San Diego, La Jolla, California, United States of America; RIKEN Genomic Sciences Center, Japan

## Abstract

Normal variation in gene expression due to regulatory polymorphisms is often masked by biological and experimental noise. In addition, some regulatory polymorphisms may become apparent only in specific tissues. We derived human induced pluripotent stem (iPS) cells from adult skin primary fibroblasts and attempted to detect tissue-specific *cis*-regulatory variants using *in vitro* cell differentiation. We used padlock probes and high-throughput sequencing for digital RNA allelotyping and measured allele-specific gene expression in primary fibroblasts, lymphoblastoid cells, iPS cells, and their differentiated derivatives. We show that allele-specific expression is both cell type and genotype-dependent, but the majority of detectable allele-specific expression loci remains consistent despite large changes in the cell type or the experimental condition following iPS reprogramming, except on the X-chromosome. We show that our approach to mapping *cis*-regulatory variants reduces *in vitro* experimental noise and reveals additional tissue-specific variants using skin-derived human iPS cells.

## Author Summary

Most complex traits likely result from a combination of genetic polymorphisms. The normal variation in gene expression is thought to be an important contributor. In order to examine a wide range of personalized tissue types from a given individual, we developed a robust method for detecting regulatory variants genome-wide in human induced pluripotent stem (iPS) cells. By having a platform capable of mapping regulatory variants despite large biological and experimental noise, and by being able to use *in vitro* differentiation to derive multiple human tissue types, our approach should enable the identification of large numbers of regulatory variants genome-wide using minimally invasive skin biopsies from a large number of human subjects.

## Introduction

The recent advances in whole genome association studies (GWAS) have uncovered multiple genetic loci linked to common human diseases and traits. In addition to the more interpretable coding sequence changes, a large number of identified loci are in the non-coding region, suggesting that inheritable regulatory polymorphisms may play an important role [1]–[3]. Expression quantitative trait loci (eQTL) studies can reveal both *cis*- and *trans*-regulatory variants that can be mapped to a specific genetic region [4],[5]. However, it requires a large sample size to reach the statistical power necessary to observe subtle changes in gene expression due to noise, ‘batch effects’ and other confounding factors [1],[6]. Current mapped eQTL loci account for only a small fraction of the overall genetic risk for a given trait, suggesting that the weak effects from multiple genetic loci may play an important role.

Although eQTL loci in different tissues can overlap [7]–[11], the range of cell types available for study still poses a problem since many regulatory pathways are tissue-specific [1],[12]. Given the potential of eQTL for elucidating genetic causes of complex traits and diseases, an ambitious effort has been launched to collect various tissue types from a large number of individuals (i.e. Genotype-Tissue Expression project). However, the existing approaches to tissue sampling, including the use of surgical and tumor specimens, are complicated by social, medical and legal issues in addition to artifacts associated with tissue collection and processing [1]. In addition, it is difficult to follow up with a functional assay in the same individual and evaluate the biological effect of regulatory variants in the absence of a viable experimental system (i.e. cell lines).

Induced pluripotent stem cells [13]–[16] can be derived from skin, hair or blood [17],[18], using transduction of reprogramming factors (i.e. OCT4, SOX2, KLF4 and MYC). They can be used to derive a number of tissues and cell types *in vitro* without resorting to invasive biopsy, and differentiation of iPS cells can theoretically allow for tissue-specific eQTL studies. However, the difficulty in observing pure and/or consistent *in vitro* differentiation can result in significant experimental variability and mask subtle regulatory variants given the practical limits on the sample size. An alternative approach may be to compare the expression level between two heterozygotic parental genes using ‘reporter’ SNPs (expression SNPs) in the exon [19]–[26]. Allele-specific gene expression (ASE) results from *cis*-regulatory differences in transcription (i.e. upstream activating sequences, DNA methylation, core promoters) or processing (i.e. alternative splicing, miRNA) [27],[28]. As such, the ASE ratio can control for the effect of experimental variations on gene expression, which function predominantly in *trans* [29],[30].

Here, we used padlock probes and high-throughput sequencing for digital RNA allelotyping to map tissue-specific expression regulatory variants in human iPS cells and their derivatives and showed that allele-specific expression analysis could overcome experimental noise and artifacts. Current approach will allow *in vitro* experiments on individualized iPS cell lines to map additional tissue-specific and context-dependent regulatory variants.

## Results

### Derivation of iPS cells from Personal Genome Project volunteer skin fibroblasts

**Figure 1:**
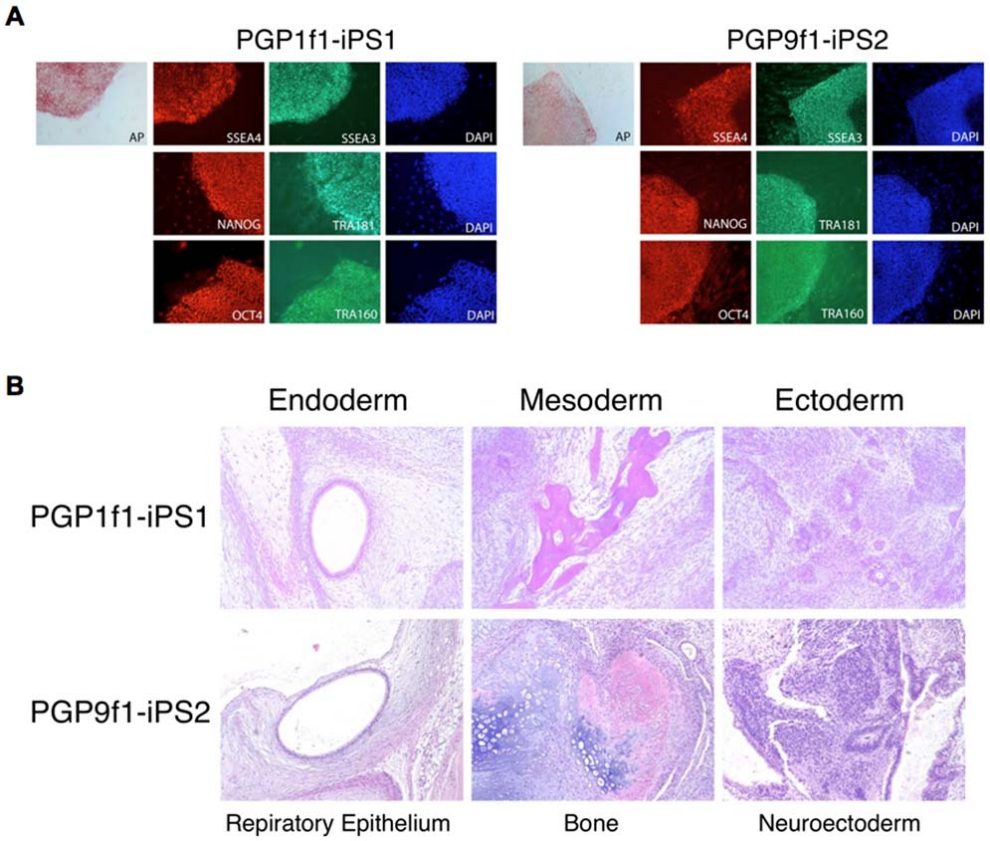
PGP induced pluripotent stem cells. (A) We derived 2–3 iPS cell lines from two biopsy sites in each individual. iPS cells expressed molecular pluripotency markers (SSEA4, SSEA3, Tra1-60, Tra1-81, NANOG and OCT4) and stained for the alkaline phosphatase activity. (B) When injected into immune-deficient mice, iPS cells formed a teratoma, containing normal tissues from all three germ layers, including respiratory epithelium (endoderm), bone (mesoderm) and neuroectoderm (ectoderm).

The Personal Genome Project (PGP) is a repository for pre-consented phenotype and genetic data as well as cell lines, including iPS cells. We derived primary skin fibroblast lines from two participants in the Personal Genome Project (PGP), using two partial depth skin biopsy samples obtained from both arms (Bx1 and Bx2). Clonal populations of PGP1 and PGP9 primary skin fibroblasts (named PGP1Bx1F and PGP9Bx1F) were isolated by routine subcloning. Non-clonal populations of primary fibroblasts (named PGP1Bx2F and PGP9Bx2F) were derived from a second biopsy site. The PGP1 and PGP9 fibroblast populations were transduced with retrovirus expressing pluripotency reprogramming factors (OCT4, SOX2, KLF4 and MYC) [31]. The isolated iPS clones expressed pluripotency markers (Figure 1A) and formed tetratomas containing normal derivatives of all three germ layers (Figure 1B), confirming their functional pluripotency.

### Padlock probes enable accurate and quantitative discrimination of alleles

**Figure 2:**
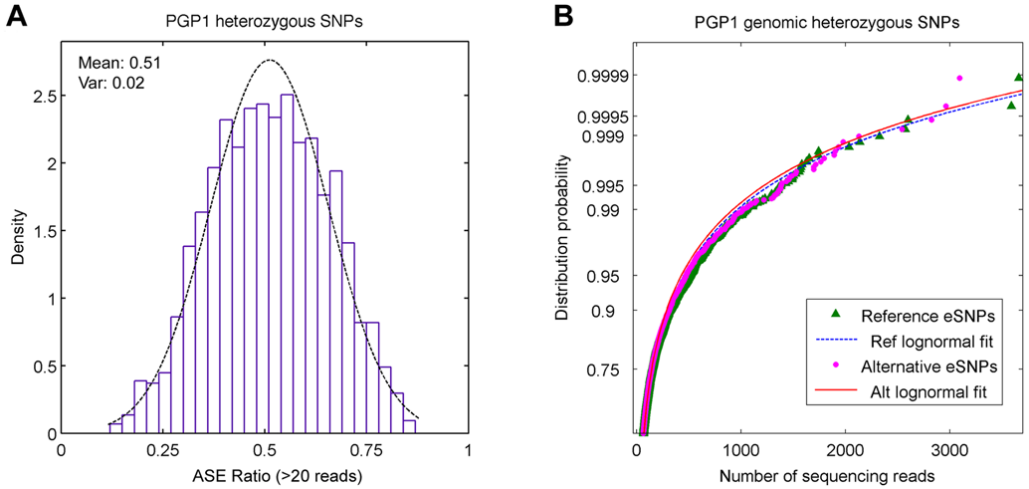
Distribution of heterozygous genotyping calls. (A) Padlock probes were used to capture approximately 19,000 SNPs from the genomic DNA. The number of mapped heterozygous reads (1.2 million) was then used to calculate the allelic ratio (reference:alternative) in 4,721 SNPs. The observed distribution of ASE ratios was symmetrically distributed around a mean of 0.51, suggesting little sequencing and/or mapping bias. (B) The heterozygous sequencing reads for both reference and alternative alleles were binned according to the observation counts. Their distribution probability was then plotted against the sequencing read counts, showing a nearly identical distribution pattern.

In order to harness the accuracy of high-throughput sequencing for quantitative allele-specific RNA analysis, we designed padlock probes targeting 27,000 common exonic SNPs (minor allele frequency > 0.07), representing 10,345 unique genes, based on the hg18 annotation (UCSC Genome Browser) (Table S1). The padlock probes were synthesized on an Agilent array in a massively parallel manner, and they were then PCR amplified and processed to generate single-stranded DNA molecules [19],[32]. The pool of single-stranded padlock probes was annealed to the double-stranded cDNA and/or the genomic DNA, followed by a 9-bp fill-in and ligation reaction to circularize the annealed probes [33],[34]. The circularized products containing the captured sequence were amplified and sequenced on Illumina GAII. On average, we obtained 6.4±2.0 million sequencing reads per sample, and we were able to map 69.8±17.2% of the reads against the RefSeq sequences used for the padlock probe design (Table 1). Approximately 19,000 (70.4%) out of 27,000 SNPs were covered at least 20 times with a mean coverage of 250 reads for each SNP, of which 25% were heterozygous calls (Table 2). Genotyping calls made using Affymetrix 500K and digital allelotyping showed a concordance rate of 98% for >20x coverage and 99% for >50x coverage (Table 3). Among the heterozygous SNPs, the ratio between reference and alternative alleles was symmetrically distributed around 0.51 (Figure 2A), and the distribution of sequencing reads was nearly identical between the two alleles (Figure 2B), suggesting little or no bias in capturing and mapping the reads.

**Table 1:** Illumina GA sequencing summary.

*N* = 25 runs	Mean	STD
Sequencing reads	6,405,521	1,983,217
Mapped reads	4,528,960	1,916,467
% mapped	69.8%	17.2%

**Table 2:** Padlock capture statistics.

SNP calling	PGP1 genomic DNA	PGP9 genomic DNA
Sequencing reads	6,891,462	6,473,738
Mapped reads	3,114,422	4,677,151
% mapped	45.2%	72.2%
SNPs called	19,013	19,175
Unique genes	8,708	8,844
Mean SNP coverage	255	251
Heterozygous SNPs	4,721 (24.8%)	4,829 (25.2%)

**Table 3:** Genotyping concordancy.

SNP coverage (# of SNPs examined)	Affy 500K Concordancy PGP1	Affy 500K Concordancy PGP9
All (n = 3,527)	96.5%	96.3%
>20 reads (n = 2,905)	97.9%	98.3%
>50 reads (n = 2,288)	98.6%	99.1%
>100 reads (n = 1,671)	99.1%	99.3%
>150 reads (n = 1,266)	99.2%	99.6%

### Allele-specific expression measurement from the cDNA using padlock probes

**Figure 3:**
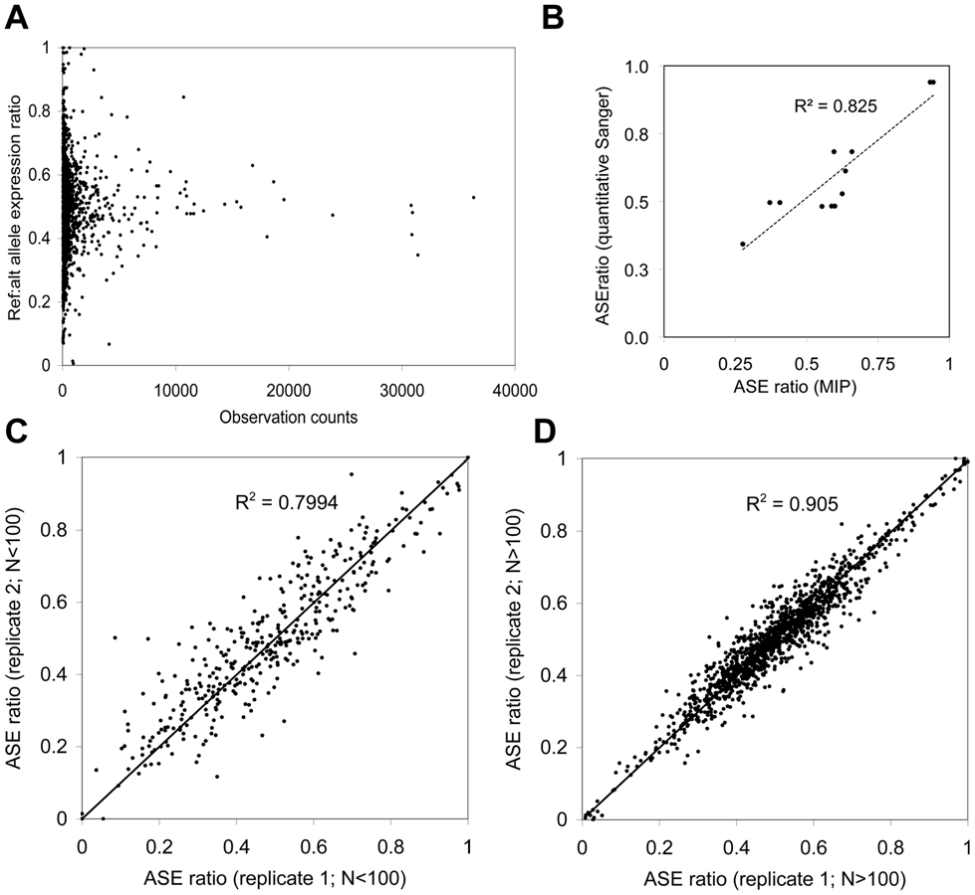
Noise and reproducibility in padlock-based ASE measurements. (A) The ASE ratio measurement from the PGP1 fibroblast cDNA (1,021 SNPs) was plotted as a function of the total number of mapped reads (reference + alternative alleles), demonstrating a relationship between the ASE ratio variance and the read count. (B) Twelve random SNPs were examined in PGP1 fibroblasts and lymphocytes using quantitative Sanger sequencing, in which the height of each sequencing trace was used to calculate the allelic ratio in the cDNA. These values were then compared against the ASE ratio determined using padlock probes. (C) The correlation among the ASE ratios with less than 100 observations remained high (R^2^ = 0.799) in technical replicates (PGP1 EB7b and EB7c). (D) Among the ASE ratios with more than 100 observations, the correlation improved to R^2^ = 0.905.

For RNA allelotyping, we amplified the singled stranded cDNA from 50 ng total RNA using linear displacement amplification (NuGen) and generated the double stranded cDNA using random hexamer priming (Invitrogen). We confirmed that the padlock probes captured both + and - strands with a similar efficiency, 51.6% and 48.4% respectively (Table 4). Typically, we observed ∼1,300 (25%) heterozygous expression SNPs out of ∼5,200 total expression SNPs. As expected, large ASE deviations were associated with SNPs having a small number of reads (<100), indicating the presence of biological and/or technical noise (Figure 3A). However, the allele-specific expression ratio was highly reproducible between the total RNA replicates (R^2^ = 0.7994 with <100 reads and R^2^ = 0.905 with >100 reads) (Figure 3C and 3D). In order to validate our method, we compared digital RNA allelotyping to quantitative Sanger sequencing, which showed a high correlation between the two methods among the 12 heterozygous expression SNPs in PGP1 samples (R^2^ = 0.825) (Figure 3B).

**Table 4:** Strand-specific padlock capture efficiency.

Strand	Targeted	SNPs (gDNA)	SNPs (cDNA)
−	13,425 (49.7%)	9,346 (49.2%)	2,974 (48.4%)
+	13,575 (50.3%)	9,668 (50.8%)	3,168 (51.6%)
Total	27,000	19,014	6,142

**Figure 4:**
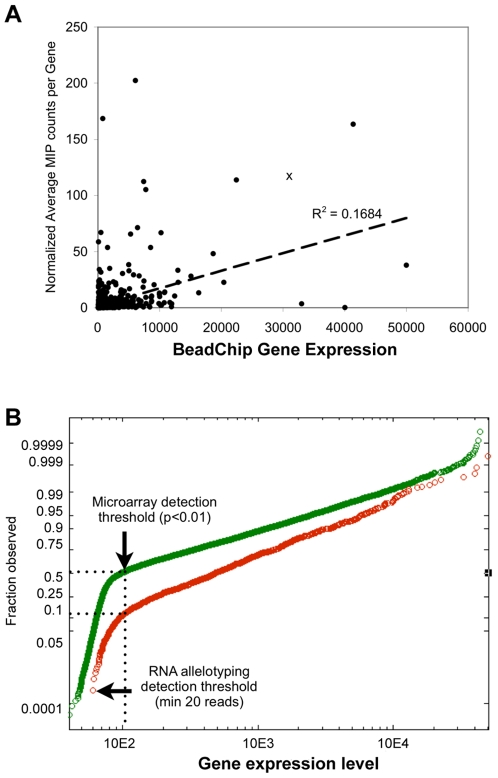
Effect of the gene expression level on ASE. (A) We examined 1,124 genes in PGP1 fibroblast cDNA shared between Illumina's BeadChip Ref-8 V3 and our padlock probe set. To normalize for the probe-specific differences in capture, the cDNA capture reads were divided by the number of reads obtained from the genomic DNA. For those genes that were targeted by padlock probes more than once, we averaged the number of normalized reads from each probe. We generated a plot of the relative gene expression from BeadChip versus the normalized average capture counts, demonstrating only a weak correlation between the level of gene expression and the frequency of padlock capture. (B) We then examined the distribution of the gene expression level detected on BeadChip (green) versus those captured by padlock probes (red). Of the 18,630 genes, approximately 50% did not reach the detection criteria (*p*-value <0.01) on BeadChip. On the other hand, 14% of all digital RNA allelotyping calls were made using these ‘undetectable’ rare transcripts.

We then asked whether the total number of reads for each SNP might reflect the gene expression level. We compared the mean number of sequencing reads from probes targeting the same transcript and normalized the values against the number of sequencing reads from the genomic DNA. We then compared these values against the relative gene expression levels as measured by Illumina BeadChip Human Ref-8, revealing only a weak correlation (R^2^ = 0.1684) (Figure 4A). We also asked whether we were capturing only those genes that were highly expressed. When we compared a list of genes captured using our method and compared it to their relative gene expression level, 159 out of 1124 (14%) captured SNPs were associated with the genes below the detection limit on the BeadChip platform (Figure 4B). These results suggested that digital RNA allelotyping was capable of detecting rare transcripts and that the absolute read counts did not necessarily reflect the overall gene expression level, possibly due to differences in probe hybridization, abundance and/or amplification.

### Individual- and tissue-specificity of allele-specific expression

In our previous study, we showed that human fibroblasts, lymphoblastoid cell lines and primary keratinocytes all demonstrated tissue-specific ASE (4.3–8.5% of heterozygous SNPs), using a different probe library design (CES22k-3.2) [19]. When adjusted for the false discovery rate in biological replicates, the percentage of SNPs with tissue-specific ASE was between 2.3–6.5%. Using a new probe design (CES27k-9bpV3), we looked for tissue-specific ASE in PGP1 fibroblasts and lymphoblastoid cell lines (Dataset S1 and Dataset S2). We observed that 3.8% (31/807) of the SNPs showed tissue-specific ASE reproducibly in both replicates. Between iPS clones and primary fibroblasts, the number of reproducible tissue-specific ASE loci increased to 9.8% (107/1091), while it was 6.9% (71/1036) between iPS cells and embryoid bodies (EBs) (Table 5). These findings suggested that up to 10% of ASE showed reproducible tissue-specificity and that they were more numerous in complex and/or heterogeneous tissue samples.

**Table 5:** Tissue-specific ASE calls (χ^2^>6.64).

Cell type comparison replicates	Shared hetSNPs	tsASE calls (%)
PGP1bx2F1:PGP1L2	808	14.4%
PGP1bx2F2:PGP1L3	1307	16.8%
Overlap	807	3.8%
PGP1bx2F1:PGP1bx2_iPS1	1186	31.5%
PGP1bx2F2:PGP1bx2_iPS6	1174	24.9%
Overlap	1091	9.8%
PGP1bx1_iPS1a:EB7	1049	26.2%
PGP1bx1_iPS1c:EB7	1309	19.2%
Overlap	1036	6.9%

**Figure 5:**
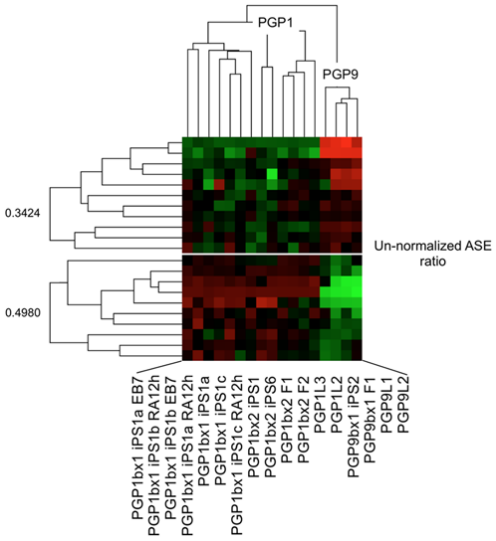
The ASE ratio alone reveals tissue- and person-specificity of regulatory differences. Of the 695 heterozygous expression SNPs commonly shared between PGP1 and PGP9, we selected 186 expression SNPs that were detectable in 17 out of 17 cDNA samples (three samples with a very low sequencing depth were excluded). Using log2-transformed ASE ratios derived directly from the sequencing counts, we performed hierarchical clustering of samples and expression SNPs (green: reference allele; red: alternative allele). We identified highly individual-specific clusters from the ASE ratio alone in the absence of any genotyping data. In addition, most tissue-specificity relationships were determined correctly from the ASE ratio.

In order to explore the relationship of ASE ratios across a wide range of tissue types, we used 186 expression SNPs that were universally present in multiple cell types from PGP1 and 9 and hierarchically clustered them using un-normalized ASE ratios directly (Figure 5). The sample correlation between the biological replicates was 0.983 (PGP1Bx2 F1 and F2) and 0.987 (PGP1Bx1 iPS1a and iPS1c), while the correlation between primary fibroblasts and lymphoblastoid cells was 0.980 (PGP1Bx2 fibroblasts versus lymphocytes). The differentiated PGP1Bx1 iPS1 derivatives were related to each other with a lower correlation of 0.969. In contrast, the ASE ratio between PGP1 and PGP9 samples had a correlation of only 0.542. We have shown previously that genetic similarity was highly correlated with allelic ratio similarity (R^2^ = 0.91) [19], and the current result confirmed this conclusion and further suggested that allele-specific expression from human iPS cells were remarkably similar to other cell types from the same individual, despite differences in their epigenetic states [35].

**Figure 6:**
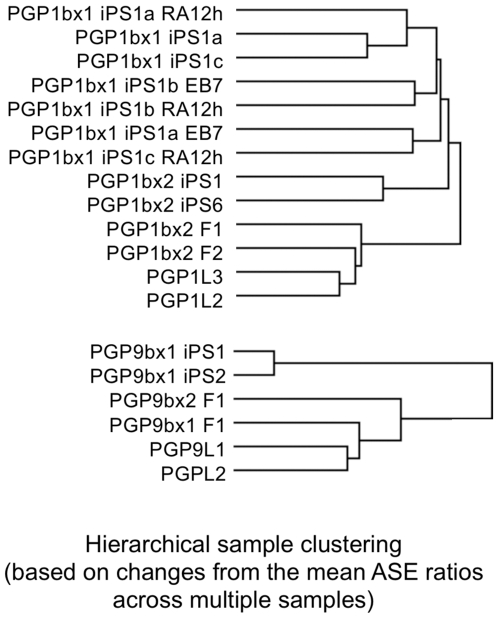
Normalized tissue-specific variations in ASE. The 186 expression SNPs were then normalized to reduce both genotyping and probe-specific biases. First, direct allelic ratios from the cDNA were normalized by those from the genomic DNA. Second, we calculated the mean ASE ratio across all samples derived from each individual and used the distance from the mean for hierarchical clustering. The normalized ASE predicted the sample relationship and the tissue-specificity with high accuracy, confirming the presence of tissue-specific regulatory expression variants.

We then normalized direct allelic ratios from the cDNA with those from the genomic DNA in order to reduce probe-specific effects on ASE measurements. To correct for a normalization bias, we calculated the mean ASE ratio across all the samples and used the distance from the mean for hierarchical clustering (Figure 6). Using the relative change in the ASE ratio across multiple cell types, we observed a consistent correlation between fibroblasts (0.31 correlation), lymphocytes (0.39 correlation) and iPS cells (0.24 correlation), while the sample correlation of fibroblasts versus lymphocytes and iPS cells was 0.27 and −0.0093, respectively. Finally, the correlation coefficient between the PGP1 and PGP9 samples was −0.26, indicating a significant difference between the two individuals. From these results, we concluded that the structure of *cis*-regulatory variants was largely genotype-dependent and that the allelic architecture in gene expression changed to a much smaller degree from cell type to cell type.

### Defining discrete ASE loci associated with regulatory variants

**Figure 7:**
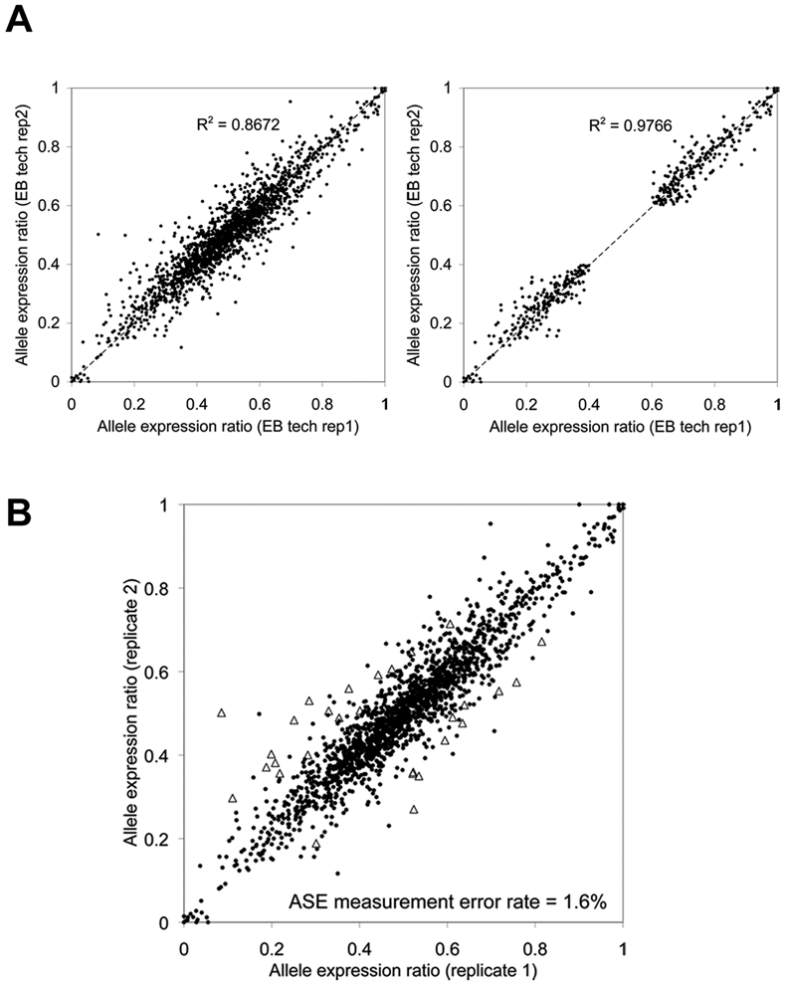
Estimation of the error rate in ASE mapping. In order to call a specific locus as being associated with a potential *cis*-regulatory variant, we used a χ^2^ -test to select the expression SNPs showing greater than a 60:40 allelic imbalance as well as having relatively higher read counts. (A) After making such confidence calls (‘mapping’) for each SNP, the reproducibility of ASE ratio measurements increased to R^2^ = 0.9802. (B) We prepared two sequencing libraries from the same pool of total RNA (technical replicates). In order to estimate the error rate in ASE calling/mapping overall, we compared the ASE ratio in these replicate libraries using a χ^2^ -test. We found that 1.6% of the statistically significant ASE calls were due to the sample processing and/or measurement error.

Strictly speaking, the ASE ratio was a quantitative measure that reflected the relative abundance of different RNA alleles. However, any detectable differences in ASE alone could also be used as an indicator of functional regulatory variants nearby. In order to assign a confidence score to ASE-mapped genes, we used a chi-squared test (cDNA-to-genomic DNA alleles; χ^2^>6.64). Since miniscule ASE could be called ‘significant’ solely due to the large number of sequencing reads, we required that the ASE ratio be >0.60 or <0.40. Therefore, our digital ASE calls addressed whether a *cis*-regulatory variant could be confidently mapped to a gene locus, not whether ASE showed a biologically meaningful allelic imbalance. When examining 427 digital ASE-positive SNPs out of 1822 total SNPs in technical replicates, the correlation coefficient of ASE ratios increased from 0.8672 to 0.9766 (Figure 7A), suggesting that much of the measurement noise had been eliminated due to a large number of observations. Using technical replicates, we also estimated the false discovery rate of digital ASE calls to be 1.6% (Figure 7B), and when all the samples were adjusted for the false discovery rate, 27±4.7% of the heterozygous expression SNPs were ‘confidently’ mapped in any given sample (Table 6). In order to show that digital ASE calls did not depend solely on the number of observations, we compared digital ASE-positive and negative calls and looked at the number of cDNA and genomic DNA reads as well as the average ASE deviation. We observed that the number of cDNA and genomic DNA reads were ∼45% higher, whereas the average allelic ratio deviation was ∼250% higher in the ASE-positive calls (Table 7). We also examined the ASE calls between PGP1 and PGP9 in order to see if they reflected the difference in allele-specific expression (Dataset S1 and Dataset S2). While the allelic deviation was ∼90% higher in the ASE-positive calls, the number of genomic DNA reads was also ∼120% higher. These results indicated that our method for mapping ASE-associated regions was influenced by all three parameters, as expected.

**Table 6:** Summary of ASE mapping calls.

Exp.	Samples	# reads	# mapped	% mapped	# het SNPs	# ASE	adj % ASE
1	PGP1 iPS1a	2949194	1506422	51.08%	1,125	293	24.5%
	PGP1 iPS1b	5315304	407556	7.67%	398	78	18.0%
	PGP1 iPS1c	5554985	2356156	42.42%	1,513	359	22.1%
2	PGP1 EBa	6510425	3518434	54.00%	1,495	545	34.9%
	PGP1 RAa	6850424	4603542	67.20%	1,691	523	29.3%
	PGP1 Ebb	7248277	5850697	80.70%	1,932	623	30.7%
	PGP1 RAb	5577200	3779730	67.80%	1,589	520	31.1%
	PGP1 EBc	7063120	5365076	76.00%	1,849	546	27.9%
	PGP1 RAc	4877544	2743289	56.20%	1,310	448	32.6%
3	PGP1 L2	3925506	3239704	82.53%	816	183	20.8%
	PGP9bx1 F1	2333798	1898168	81.33%	1,021	297	27.5%
	PGP9bx1 iPS1	3785125	3192330	84.34%	1,100	262	22.2%
	PGP9bx1 iPS2	3307449	2751313	83.19%	1,117	259	21.6%
	PGP1bx2 F1	8214253	6325717	77.01%	1,519	391	24.2%
	PGP1bx2 iPS1	8022045	6196964	77.25%	1,611	584	34.7%
	PGP1bx2 iPS6	7107819	5859125	82.43%	1,629	500	29.1%
	PGP9bx1 F2	9717319	7747988	79.73%	1,368	420	29.1%
	PGP9 L1	9299960	7160842	77.00%	1,199	314	24.6%
	PGP9 L2	8774562	6576997	74.96%	1,439	451	29.8%
4	PGP1bx2 F2	7390237	5390680	72.94%	1,420	344	22.6%
	PGP1 L3	6153115	4774427	77.59%	1,518	472	29.5%

(χ^2^ >6.64; >60% allelic imbalance. Estimated false discovery rate  = 1.6%)

**Table 7:** Observation frequency and ASE calling.

PGP1	“ASE = 1” (n = 468)	“ASE = 0” (n = 1,468)
Average cDNA count per probe	747.8	729.3
Average gDNA count per probe	498.6	295.9
Ave. deviation from bi-allelic exp	0.2569	0.1087

**Figure 8:**
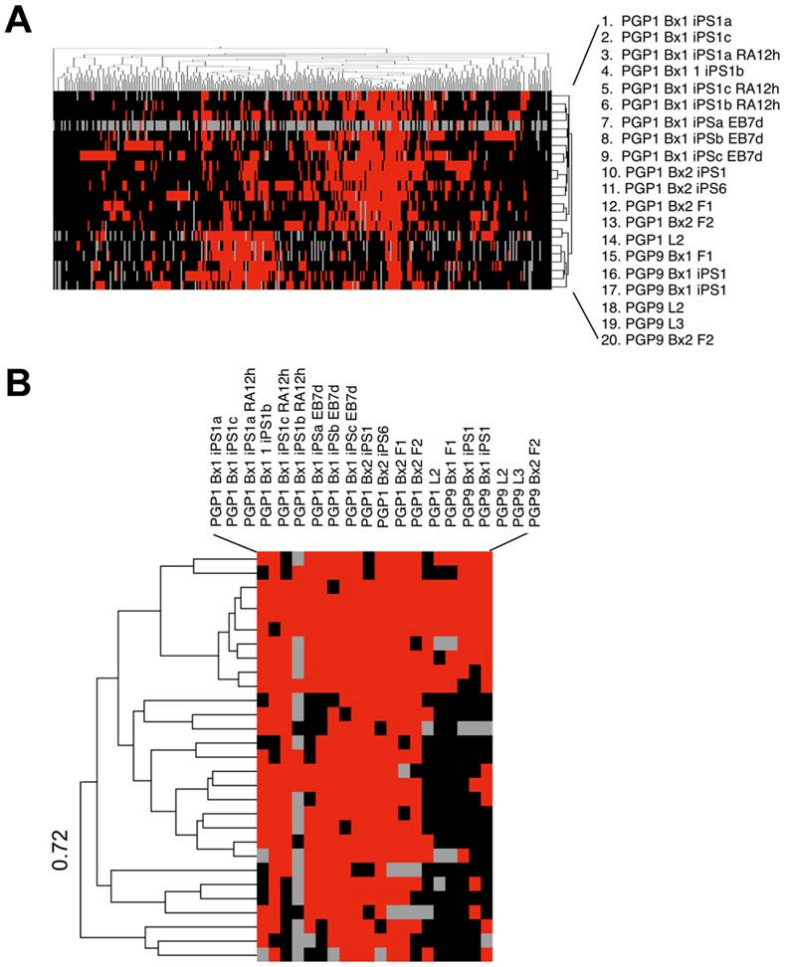
Visualizing the mapped ASE loci in multiple tissue types. (A) In order to see if mappable ASE loci depended on the tissue type being examined, we examined 317 expression SNPs present in at least 16 out of 20 samples. Mapped ASE loci (red), unmapped ASE loci (black) and missing ASE loci (gray) were hierarchical clustered to reveal tissue-specific sample clustering. (B) When one of the most significant cluster mapping nodes (0.72 correlation) were examined, we found 29 expression SNPs that were consistently mapped in >80% of the cell types derived from PGP1.

In order to visualize tissue-specific ASE loci associated with high confidence scores, we examined 1522 heterozygous expression SNPs in 20 PGP1 and PGP9 samples, out of which 317 SNPs were shared among at least 80% of the samples. When these digital ASE calls were hierarchical clustered, there were able to discriminate different tissue types and individuals (Figure 8A). A possible explanation of why digital ASE calls reflected tissue-specificity was that higher tissue-specific expression resulted in higher cDNA observation counts. However, we previously demonstrated that there was no appreciable difference in the number of cDNA reads between ASE-positive and -negative calls in a variety of tissues (Table 7). In addition, the average number of sequencing reads correlated poorly with the absolute gene expression level (Figure 4A), suggesting that the differences in read counts alone did not explain tissue-specific ASE mapping. We also examined individual-specific ASE-positive clusters with the sample correlation of 0.7223 in PGP1 (29/317). Interestingly, a large fraction of PGP1-specific clusters were characterized by consistent ASE calls across all cell types (Figure 8B), indicating that approximately 1/3–1/2 of the mapped *cis*-regulatory variants were cell type and context-independent.

### Mapping tissue-restricted ASE loci using iPS cells

So far, we attempted to map *cis*-regulatory variants using the gene transcripts that were universally present among various cell types and found that up to 10% of the genes might be influenced by tissue-specific regulatory variants. However, we expected that other *cis*-regulatory variants would only be detected using tissue-specific transcripts. In order to capture these variants, we compared different cell types with a similar sequencing depth (5.3–7.4 million reads) and counted the number of ASE-positive calls that were specific to that tissue. We were able to examine between 1,500 to 1,900 heterozygous expression SNPs in primary fibroblasts, immortalized B-lymphocytes, iPS cells and iPS-derived embryoid bodies (EBs) from PGP1 (Table 8). The number of expression SNPs unique to each cell type was 34 (2.2%) and 49 (3.2%) for fibroblasts and lymphocytes, respectively. In contrast, we observed 126 (7.8%) and 287 (14.9%) tissue-restricted expression SNPs in iPS cells and EBs, respectively. This suggested that iPS cells and EBs expressed many transcripts absent in primary cell lines. In addition, we found that the percentage of ASE-positive SNPs was generally lower in fibroblast- and lymphocyte-specific transcripts (∼24%) as compared to iPS and EB-specific transcripts (∼38%). Overall, the number of ASE-positive loci mapped using primary fibroblasts alone was 391, which increased to 562 (44% increase) using iPS cells and limited *in vitro* differentiation. We estimated that more than 12% of all heterozygous SNPs were associated with ‘mappable’ functional regulatory variants using our approach. We expect this number to increase when other differentiated cell types are examined.

**Table 8:** ASE-associated with tissue-restricted transcripts.

Samples	Mapped reads	hetSNP	Tissue-restricted hetSNP	Tissue-restricted ASE	%ASE in tissue-specific transcripts
Fibroblast	6,003,854	1,519	34	8	23.5%
Lymphocytes	7,381,108	1,518	49	12	24.5%
iPS	5,818,957	1,611	126	48	38.1%
EB	5,343,178	1,932	287	111	38.7%

### iPS reprogramming shows the inversion of ASE on the X chromosome

**Figure 9:**
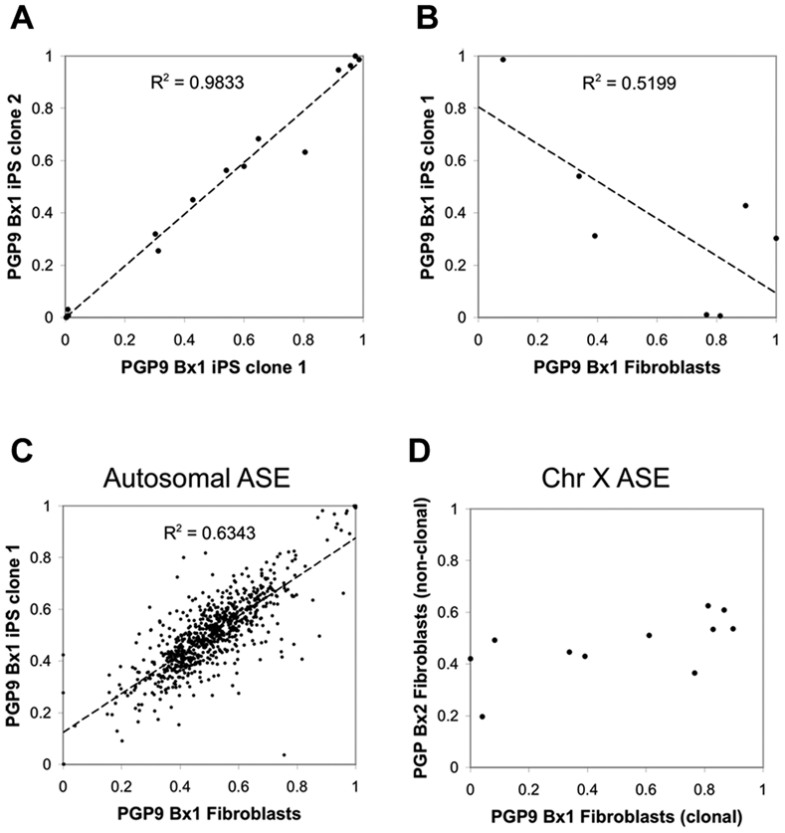
Changes in ASE reflects X-chromosomal silencing during iPS reprogramming. (A) In the two iPS clones derived from PGP9, the X-chromosomal ASE ratio was highly reproducible, in which 7 out of 14 SNPs escaped complete silencing (R^2^ = 0.9833). (B) When compared to their parental primary fibroblasts (clonally derived PGP9Bx1F1), we observed that the X-chromosomal ASE ratio was now inversely proportional (R^2^ = 0.5199), strongly suggesting the re-activation of the random silencing mechanism. (C) Autosomal genes in the same pair showed a positive correlation (R^2^ = 0.6343). (D) When PGP9Bx1F1 (clonally derived) was compared to PGP9Bx2F1 (non-clonal), the X-chromosomal ASE ratio confirmed the clonality of PGP9Bx1F1 used for iPS reprogramming.

Dosage compensation in mammalian somatic cells is achieved by randomly silencing one of the transcriptionally active X-chromosomes [36]. Random X-inactivation in mouse ES cells is tightly coupled to cell differentiation and the silenced X-chromosome can be re-activated by somatic nuclear transfer [37]. In order to determine how ASE might be affected by re-activation of the silenced X-chromosome after iPS reprogramming, we used a clonal population of female primary fibroblasts to generate two iPS cell lines (PGP9Bx1 iPS1 and PGP9Bx1 iPS2). We then examined 66 heterozygous expression SNPs that were present on the X-chromosome. We observed 14 genes (21%) that were expressed and captured in the two iPS cell lines from PGP9. The ASE ratios of these genes were highly reproducible (R^2^ = 0.98), including 6 out of 14 SNPs (42%) showing a near mono-allelic preference (Figure 9A). We also observed that eight X-chromosomal expression SNPs were shared between PGP9Bx1 F1 and PGP9Bx1 iPS2. Surprisingly, their ASE ratios were proportionately reversed with a negative linear correlation of R^2^ = 0.52 (Figure 9B). In contrast, the autosomal ASE ratios in the same pair of cell lines demonstrated a positive linear correlation (R^2^ = 0.63) (Figure 9C). When we examined a polyclonal population of primary fibroblasts (PGP9Bx2F1), their X-chromosomal ASE ratios were near 0.5, likely due to the population averaging of random X-chromosomal inactivation (Figure 9D). These results indicated that both complete and partial inversions of X-chromosomal ASE ratios occurred during iPS reprogramming and that our method was sensitive and robust enough to detect true changes in allele-specific expression due to reasons other than *cis*-regulatory polymorphisms.

### *In vitro* iPS differentiation reveals the inversion of autosomal ASE

**Figure 10:**
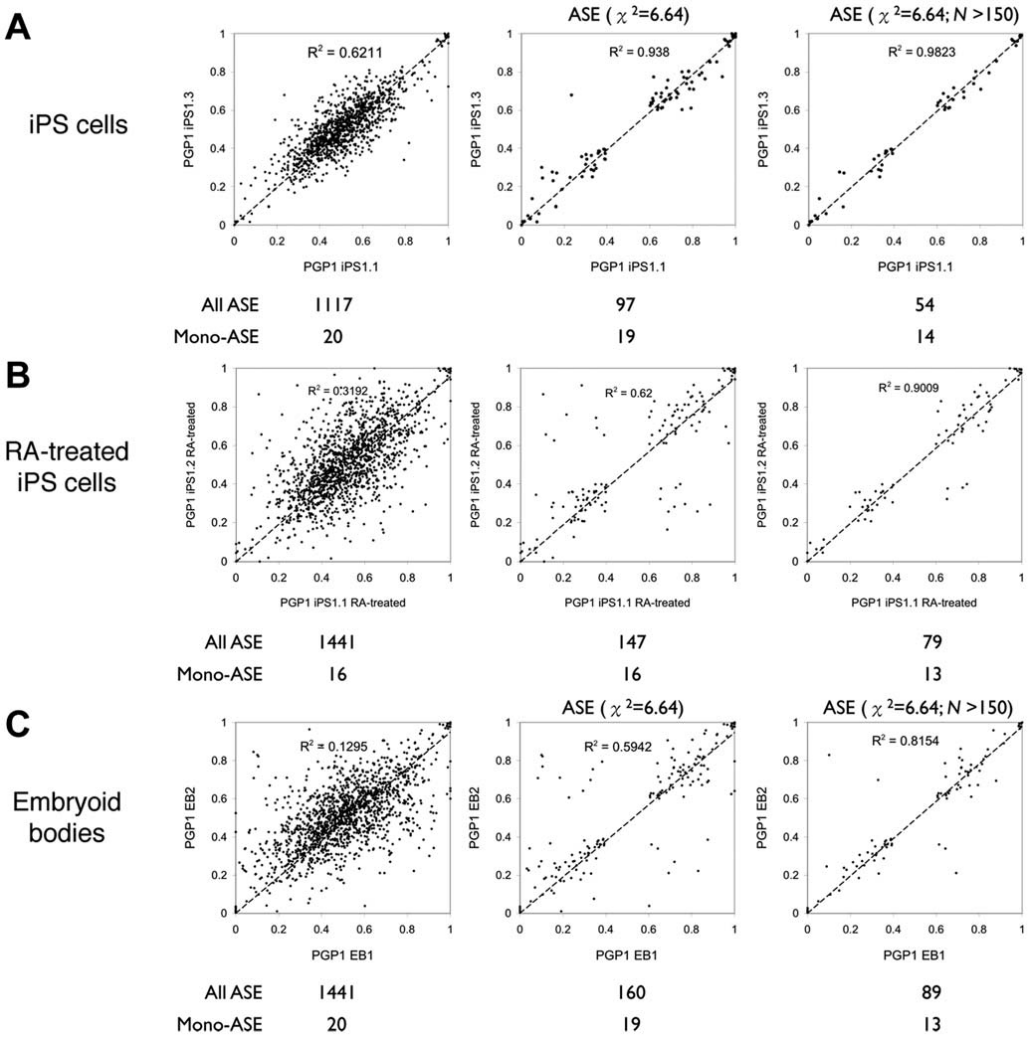
Variations in the mappable ASE loci with *in vitro* iPS differentiation. iPS cells (A), retinoic acid (RA)-treated iPS cells (B), and embryoid bodies (C) were compared to another iPS clone from the same individual pair-wise. Two different types of statistical threshold were used to categorize ‘mappable’ ASE loci. Mono-allelic expression is defined as having an allelic preference of >90%. *N* is the number of observation from cDNA samples. Retinoic acid was added directly to newly suspended iPS colonies growing on a low-attachment plate for 12 hours. Embryoid bodies were cultured the same way without retinoic acid for 7 days.

**Figure 11:**
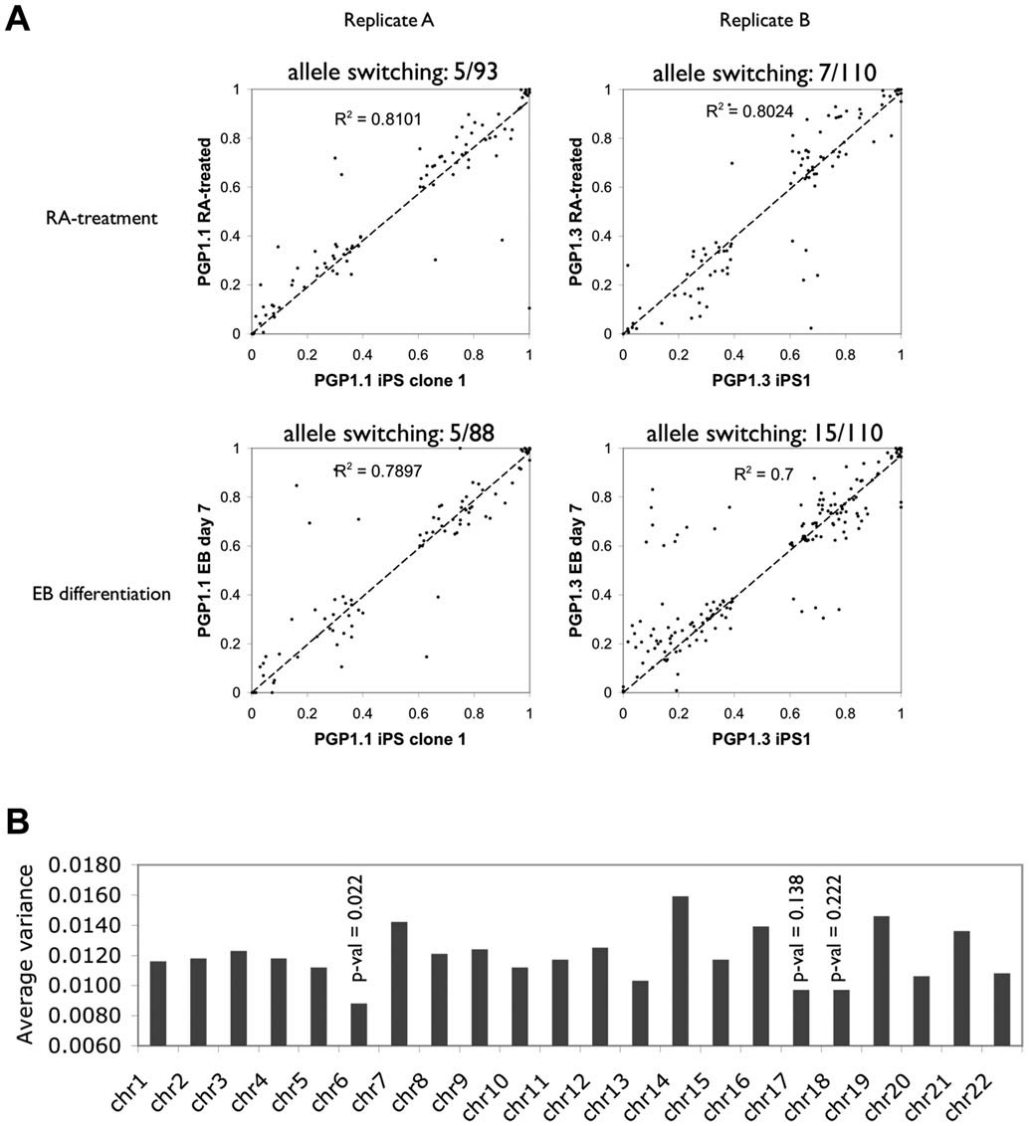
Changes in ASE during iPS differentiation. (A) A biological replicate of PGP1Bx1 iPS1 cells were used for *in vitro* differentiation, using 100-µM trans-retinoic acid for 12 hours or embryoid body formation for 7 days. ASE loci with high confidence scores are shown. The expression SNPs in the upper left and the lower right quadrant represent those that have significantly altered their preference of parental alleles (5–13%). (B) Variations in ASE across six differentiated samples (3 RA-induced and 3 EBs) were analyzed according to their chromosomal locations. The ASE ratio variance from Chromosome 6 was compared to the ASE ratio variance from all other chromosomes.

We then examined ASE in undifferentiated and differentiating iPS cells. When considering only the ASE-positive SNPs, we observed that the correlation between iPS biological replicates (R^2^ = 0.94) was similar to that of technical replicates (R^2^ = 0.98) (Figure 10A). When iPS cells were treated with 100-µM *trans*-retinoic acid for 12 hours, the ASE ratio showed a reduction in correlation between replicates (R^2^ = 0.62), likely due to the heterogeneity of the colony size and the differentiation environment (Figure 10B) [38]. When the iPS cells were further differentiated into embryoid bodies (EBs) for 7 days, we similarly observed a reduction of correlation between replicates (R^2^ = 0.59) (Figure 10C). We also found that up to 5–13% of the ASE-positive expression SNPs switched the allelic preference during transient and long-term iPS differentiation (Figure 11A), indicating that parental isoforms could be alternately expressed during developmental transitions. While this phenomenon could be due to random stochastic noise, we showed that the ASE ratio was highly reproducible between biological and technical replicates, even among the rare gene transcripts falling below the traditional detection limit. This suggested that ASE switching was due to the biological heterogeneity of stem cell differentiation and not random measurement noise alone. Finally, changes in autosomal ASE did not affect all chromosomes equally during iPS differentiation (*N* = 6 samples). We observed that Chromosome 6 displayed lower ASE variance that was statistically significant (p-value: 0.022), possibly due to the amount of stable gene imprinting present on Chromosome 6 (Figure 11B). This observation also supported the idea that the variability in ASE during iPS differentiation was not solely due to random noise.

## Discussion

Studying subtle and/or normal variations in gene regulation requires a sensitive and robust method for measuring true genetic effects. Such effects should be measured in a wide range of human tissues, whether by using human tissue samples or *in vitro* cell culture, both of which can introduce many confounding factors and experimental artifacts. By combining alleles-specific expression analysis together with human pluripotent stem cell reprogramming, we were able to achieve both objectives with high sensitivity and reproducibility. Despite extreme variations in the cell types, the epigenetic status, cell derivation and reprogramming methods and cell differentiation protocols, we were able to detect a subtle allelic imbalance as small as 60:40 and map approximately 27% of the expression SNPs in a given cell line, of which 3–10% were tissue-specific. We also demonstrated that 1/3–1/2 of mappable ASE loci were reproducible regardless of the cell type used and that they were strongly dependent upon the genotype. We also showed that differentiated iPS cells expressed >40% more transcripts associated with ASE and that more should now be mappable using directed *in vitro* differentiation. Finally, xwe demonstrated two examples of dramatic ASE changes during X-chromosomal inactivation and during iPS differentiation, showing that our approach can successfully detect global changes in allele-specific gene regulation during development.

The reproducibility of ASE loci across many different cell types was reassuring, but it also pointed to the possibility of having a systematic bias throughout all the samples. Thus, we asked whether we could find an example of ASE changes that was both expected and biologically interpretable. We found that the X-linked ASE ratio was proportionately inversed after iPS reprogramming, including those that were partially silenced. It was known that up to 25% of the X-linked genes could escape X inactivation in human cell lines [39], and indeed, we observed 7/23 and 4/16 X-linked SNPs that were only partially silenced in PGP9 iPS cells and fibroblasts, respectively. Our study demonstrated that these genes were still influenced by X inactivation and that the effect remained proportionately similar even after random chromosomal silencing. While nuclear reprogramming has been reported to reset random X-inactivation in cloned mouse embryos [37] and in mouse iPS cells [40], it was not known whether human iPS cells reached an embryonic ground state. However, we showed that human iPS cells from clonal primary fibroblasts possessed an inverted X-chromosome inactivation pattern, suggesting that human iPS reprogramming can indeed completely erase the somatic X-inactivation memory, a property associated with the embryonic ground state.

Conceptually, allele-specific expression is a direct result of functional *cis*-regulatory mutations or variations. However, it is also caused by random stochastic events [41],[42] and gene imprinting/silencing [43] as well as allele-specific methylation [22]. Because iPS reprogramming is accompanied by a high degree of cell clonality and epigenetic changes, it offered us an unprecedented opportunity to study how allele-specific expression was affected by such factors. Using a genome-wide allele-specific expression analysis on multiple cell types derived from the same individual, our study conclusively showed that the mappable ASE loci were not dramatically affected by the cell clonality, the methylation status and/or the pluripotency reprogramming and that they were highly individual-specific. It indicated that allele-specific expression might be a good surrogate for indicating the presence of functional *cis*-regulatory variants. The next logical step will be to determine whether this mappable ASE loci are in fact inheritable and that they can combine in the offspring to produce a gene expression phenotype that is much more dramatic and biologically significant.

While it is tempting to use the ASE ratio as a quantitative trait for association mapping, most ASE loci may not produce a strong phenotype in heterozygous individuals. However, allele-specific expression may exert a more direct influence when combined with other functional variants to generate a mixture of functionally altered protein isoforms. With full diploid genome sequencing, it may now be possible to measure the frequency of allelic combinations that may produce measurable effects on the protein function as well as the signaling and/or transcriptional pathways in an allele-specific manner. Our study showed that as many as 5–13% of the mapped ASE loci changed their preference of parent-specific gene expression during early iPS differentiation and development. It will be fascinating to examine whether alternating patterns of parent-specific gene expression associated with functional coding variants can give arise to subtle variations in parent-specific cellular and tissue organization during different phases of the human development.

While the most straightforward cause of allele-specific expression is differential transcription factor binding on the promoter, other mechanisms such as alternative splicing and methylation-mediated repression may also play an important role. We are currently developing technologies for examining additional molecular features beyond gene transcription to explore allele-specific processes during gene expression and processing. While functional haploid cells and organisms have greatly enhanced our understanding of various molecular pathways in simple organisms, especially in conjunction with mutagensis screening, such approaches are not possible in higher eukaryotes such as mice and humans. However, an allele-specific readout such as ASE allows one to study the effect of haploid elements and variations in fully functional cell lines, enabling one to design experiments to dissect the phenotypic consequence using family of cell lines with different genetic combinations. Therefore, the real power of ASE and other analyses may not necessarily reside in their ability to map of regulatory variants, but to determine the mechanism of allelic combinations that can contribute to the development of a complex inheritable phenotype.

While the use of iPS cells and allele-specific expression analysis for expression trait mapping shows much promise, there are limitations to this approach. The iPS reprogramming, and the propagation and differentiation of iPS cells can be laborious and do not scale up easily. It also does not distinguish among various possible mechanisms for allele-specific expression (i.e. promoter activation, alternative splicing, sequence-specific degradation). In order to bypass these bottlenecks, we are engaged in an effort to automate cell immortalization/iPS reprogramming as well as allele-specific expression assays in order to examine a large population of human volunteers with extensive phenotype and genotype data (Personal Genome Project). Leveraging the power of full genome sequencing technology, our approach of using padlock probes will enable one to examine thousands of samples simultaneously, providing a way to explore *cis*-regulatory variants in many different tissues in thousands of living study volunteers cost-effectively. We are currently also targeting potential regulatory variants using zinc finger nuclease-mediated homologous recombination in iPS cells to alter their ASE profile and the gene expression level. This and other similar efforts to map and understand numerous functional variants in the vast stretches in the non-coding region and integrating it with experimental biology in a high-throughput manner will likely yield a potent insight into the person-specific regulation in gene expression, cellular biology and ultimately, personalized medicine.

## Materials and Methods

### Ethics statement

Personal Genome Project (PGP) obtained informed consent from human volunteers who have agreed to release both genetic and tissue samples to the research community. All protocols relating to the collection and processing of human data and samples have been approved by Harvard Institutional Review Board (IRB).

### Cell lines and tissue culture

The primary fibroblasts were maintained in 15% NCS (Hyclone) D-MEM/F12 (Gibco) supplemented with 10 ng/ml hEGF (R&D Systems), non-essential amino acid (Gibco), Pen/Strep and L-Glutamine (Gibco). The iPS cells were maintained in 20% KO-Serum (Invitrogen) KO-DMEM (Invitrogen) supplemented with 4 ng/ml bFGF (BD Biosciences), β-ME (Gibco), non-essential amino acid, Pen/Strep and L-Glutamine on a γ-irradiated MEF layer (GlobalStem).

### Generation of human induced pluripotent stem cells [31]

Briefly, pMIG containing OCT4, SOX2, KLF4 and MYC along with VSV-G and Gag-Pol vectors were transiently transfected into 293T cells. We collected retrovirus-containing medium and passed through a 0.45-micron filter unit, followed by ultracentrifugation. We added each virus at multiplicity of infection (MOI) of 5 to human primary fibroblasts (passage number <8). We found that clonally derived PGP1Bx1 fibroblasts were more difficult to reprogram, and it required SV40 large T and NANOG to achieve functional pluripotency [13]. By day 21–30 post-infection, hES cell-like flat colonies started to appear, and they were picked manually and propagated on a freshly prepared MEF layer.

### RNA isolation and amplification

The total RNA was prepared using RNeasy (Qiagen). The RNA sample was then linearly amplified and synthesized into a single-strand cDNA using a whole transcriptome amplification method (NuGen). The linearly amplified single-stranded cDNA is then converted into double-stranded cDNA fragments using random hexamers and *E. coli* DNA polymerase at 16°C for 2.5 hours. Of note, we did not observe a significant difference in read counts between the first strand and the second strand (Table 4).

### SNP capture and sequencing

Circularization was performed in 20-ul reactions containing 400 ng genomic DNA or 200 ng ds-cDNA, 0.5 pmole padlock probes (total concentration), 2U AmpLigase (Epicenter), 2U AmpliTaq Stoffel fragment (Applied Biosystems), 0.1 µM dNTP in 1x AmpLigase buffer. The reactions were incubated at 95°C for 5 minutes, 60°C for 48 hours. The reactions were then denatured at 94°C for 1 minutes, cooled down to 37°C, then digested with Exonuclease I (10U) and Exonuclease III (100U) for 2 hours at 37°C, and finally heat inactivated at 94°C for 5 minutes. Post-capturing PCR reactions were performed in 100-ul reactions including 10-ul circularization products, 0.4x SYBR Green I, 0.4 µM forward and reverse PCR primers in 1x iProof PCR master mix. The parameter for real-time PCR was 98°C 30 seconds; followed by 3 cycles of 98°C 15 seconds, 53°C 20 seconds, 72°C 10 seconds; then <15 cycles of 98°C 15 seconds and 72°C 20 seconds. We terminated the reactions when the amplification curves went up close to the plateau stage. The 154-bp amplicon was purified with a 6% TBE polyacrylamide gel (Invitrogen), and sequenced with Illumina Genome Analyzer II.

### Data analysis

We designed the padlock probes to ensure that the captured sequences are uniquely mappable to the genome using UCSC BLAT. We mapped sequencing reads (25–41 bp) to the sequences by NCBI BLAST using the word size of 8–12 depending on the read length, considering the variant site as degenerate (NCBI Short Read Archive #SRA008291.1). For any sequences that had more than one hit, we required that the second hit had an e-value 5-fold higher than the top hit. In contrast, Maq-based mapping could not handle degenerate sequences, and it was consistently biased towards the reference allele. We made genotyping calls using the “best-P” method on SNPs that were sampled at least 20 times. For each SNP we performed both the test of homozygosity (assuming the allelic ratio of (1-*e*)/*e* where *e* is the sequencing error) and the test of heterozygosity (assuming 50:50 allelic ratio), and determined the genotype based on the one that giving a higher *p*-value. We used chi-squared test to identify expressed SNPs that exhibit RNA allelic ratios significantly different from the genomic allelic ratios (see Table S1, Dataset S1, Dataset S2). Hierarchical clustering and image viewing were done on Cluster and TreeView.

## Supporting Information

Table S1 CES27k-9bpV3 padlock probe annotation file.
(16.77 MB XLS)

Dataset S1 PGP1 normalized digital allele-specific expression dataset.
(2.35 MB XLS)

Dataset S2 PGP9 normalized digital allele-specific expression dataset.
(1.45 MB XLS)
